# Chemical Food Safety of Edible Insects in the European Union: Current Evidence and Regulatory Blind Spots

**DOI:** 10.3390/toxics14090774

**Published:** 2026-08-31

**Authors:** Zsuzsa Farkas, Theodora Bernitsa, Maria do Céu Costa, József Lehel

**Affiliations:** 1 Department of Digital Food Science, Institute of Food Chain Science, University of Veterinary Medicine Budapest, István Str. 2, H-1078 Budapest, Hungary; farkas.zsuzsa@univet.hu; 2 CBIOS (Research Center for Biosciences and Health Technologies), ECTS (School of Health Sciences and Technologies), Lusófona University, Campo Grande 376, 1749-024 Lisboa, Portugal; maria.costa@ulusofona.pt; 3 Department of Food Hygiene, Institute of Food Chain Science, University of Veterinary Medicine Budapest, István Str. 2, H-1078 Budapest, Hungary; lehel.jozsef@univet.hu

**Keywords:** edible insects, chemical food safety, novel foods, heavy metals, mycotoxins, pesticide residues, processing contaminants, risk assessment, European Union regulation

## Abstract

Edible insects are increasingly promoted as alternative foods, but their chemical safety is difficult to assess because hazards may arise throughout the production chain and the regulatory framework remains incomplete. This structured narrative review synthesises current evidence on chemical hazards in edible insects and examines their interpretation within the European Union regulatory framework. The available literature indicates that rearing substrates and production environments are major sources of contamination, particularly for heavy metals, mycotoxins, pesticide residues, veterinary medicinal product residues, and persistent or emerging contaminants. However, transfer into insect biomass is highly species-, life-stage-, and compound-dependent, and metabolism, excretion, or transformation may reduce parent-compound concentrations without eliminating uncertainty regarding metabolites and other transformation products. Processing and storage may further modify the chemical profile through the formation of acrylamide, furan derivatives, lipid-oxidation products, and biogenic amines. Although the EU novel food framework provides product-specific premarket assessment, representative market-occurrence and consumption data remain limited, and generally applicable insect-specific maximum levels for contaminants have not been established, whereas pesticide residues are governed by the horizontal EU MRL framework. Chemical safety assessment should therefore adopt a full-chain, species- and product-specific approach integrating substrate control, contaminant fate, processing, storage, and consumer exposure.

## 1. Introduction

### 1.1. Background and Global Relevance of Entomophagy

The consumption of insects, or entomophagy, has a long history in human diets and remains widespread globally. However, precise estimates of the number of consumers remain uncertain, and the widely cited estimate of approximately 2 billion consumers has been considered likely to represent an overestimate [1]. More than 2000 insect species have been documented as edible, although the exact number also depends on definitions, documentation, and taxonomic identification [2]. Edible insects may be collected from the wild or produced under controlled farming conditions and consumed whole or incorporated into processed foods [3].

Edible insects are taxonomically diverse and include species from several orders such as Coleoptera, Lepidoptera, and Orthoptera. Commonly consumed examples include crickets (*Acheta* spp. and *Gryllus* spp.), silkworm pupae (*Bombyx mori*), and palm weevil larvae (*Rhynchophorus* spp.) [3,4]. Interest in edible insects has expanded beyond regions where entomophagy is traditionally practised, driven partly by their potential contribution to more sustainable food systems. Their feed conversion efficiency, comparatively low environmental resource requirements, and capacity to use certain organic side streams have supported their development as alternative protein sources [5,6,7].

### 1.2. Nutritional Relevance of Edible Insects

Many edible insect species are characterised by high nutritional value, although their composition varies considerably according to species, developmental stage, rearing conditions, substrate, and processing methods. They can provide substantial amounts of protein and lipids, together with minerals and vitamins [4,8,9,10]. Their lipid fraction may include mono- and polyunsaturated fatty acids, while commonly reported micronutrients include calcium, zinc, magnesium, B-group vitamins, and vitamin E [9,10].

This nutritional potential has encouraged the incorporation of insects into a growing range of products, including milling products, protein concentrates, snacks, bakery products, and other composite foods. However, the expansion and diversification of insect-based foods also introduce distinct chemical safety considerations. Contaminants may originate from rearing substrates and the production environment, while processing and storage may alter existing contaminants or generate additional hazards.

### 1.3. Edible Insects as Novel Foods in the European Union

Within the European Union (EU), foods that were not consumed to a significant degree by humans before 15 May 1997 are regulated as novel foods under Regulation (EU) 2015/2283 and require premarket authorisation following safety assessment [11]. Novel foods (NFs) derived from four insect species have been authorised: yellow mealworm (*Tenebrio molitor* larva), lesser mealworm (*Alphitobius diaperinus* larva), migratory locust (*Locusta migratoria*), and house cricket (*Acheta domesticus*). Authorised forms and conditions of use vary among individual NFs and include whole, frozen, dried, and powdered products [11,12].

The NF framework provides a structured, case-by-case assessment of individual NF applications and establishes specifications and conditions of use for authorised products. However, it does not itself establish generally applicable contaminant maximum levels (MLs) or pesticide maximum residue limits (MRLs) for insects. These are addressed, where applicable, through horizontal EU legislation; pesticide residues in insects fall within the scope of Regulation (EC) No 396/2005 [13].

### 1.4. Aim of This Review

The available evidence on chemical hazards in edible insects is distributed across studies of individual contaminant classes, environmental studies, research on insects used as feed, controlled exposure experiments, studies of commercial products, processing studies, and product-specific regulatory assessments. These evidence sources differ substantially in their direct relevance to commercially produced insects intended for human consumption. Considering these evidence streams in isolation may obscure the interactions among contamination sources, insect-specific uptake and transformation, post-harvest processing and storage, and final consumer exposure.

Recent reviews have summarised the nutritional value, sustainability, production, and general food safety aspects of edible insects. However, comparatively limited attention has been paid to integrating evidence on chemical contaminants with insect-specific exposure pathways, contaminant transformation, and the current EU regulatory framework.

This review synthesises international evidence on chemical food safety hazards in edible insects while focusing its regulatory analysis on the European Union. Specifically, it (i) integrates evidence across major contaminant classes within a full-chain exposure framework linking the rearing substrate and production environment to insect uptake, metabolism, processing, storage, final food products, and consumer exposure; (ii) distinguishes among evidence derived from environmental studies, research on insects used as feed, controlled experiments, commercial product studies, and novel food dossiers, and critically evaluates its relevance to human food safety; and (iii) examines how chemical hazards are addressed in European Food Safety Authority (EFSA) NF assessments and EU regulatory practice, including the distinction between product-specific specifications and generally applicable regulatory limits and the gaps in post-market occurrence data.

By linking contaminant-specific evidence with production pathways and regulatory interpretation, this review moves beyond a hazard-by-hazard summary and provides a structured basis for identifying priorities for insect-specific risk assessment, monitoring, and regulatory adaptation.

## 2. Review Scope, Evidence Identification and Synthesis

### 2.1. Review Design

This review was conducted as a structured narrative review synthesising current evidence on chemical food safety hazards associated with edible insects. It was designed as a narrative evidence synthesis rather than a systematic review. Accordingly, the literature identification was iterative and topic-specific and was not based on a single predefined search yielding a fixed set of records for formal systematic screening. The review focused primarily on chemical contaminants relevant to insects intended for human consumption, while biological, physical, and allergenic hazards were included only to provide context for the overall food safety framework. Particular emphasis was placed on the EU regulatory framework, including EFSA novel food assessments and the legislation governing edible insects and chemical contaminants.

### 2.2. Information Sources and Evidence Identification

Literature searches were conducted primarily using the Web of Science Core Collection and Scopus databases. Multiple topic-specific searches were performed rather than a single comprehensive search string, because the review covered heterogeneous contaminant classes, exposure pathways, processing-related hazards, and regulatory questions. Searches combined terms related to edible insects (e.g., “edible insects”, “insect-based food”, “novel food insects”) with contaminant-specific keywords corresponding to the topics addressed in this review, including heavy metals (e.g., cadmium, lead, mercury, arsenic), pesticide residues, mycotoxins, veterinary drug residues, persistent organic pollutants (POPs), per- and polyfluoroalkyl substances (PFAS), brominated flame retardants, processing- and storage-related chemical hazards (e.g., acrylamide, furan and its derivatives, monochloropropane-1,2-diol substances (MCPDs)), and other terms associated with chemical food safety. Search terms and combinations were adapted iteratively to the individual evidence domains and were refined where relevant terminology, contaminant classes, insect species, or regulatory issues emerged during the review process.

Greater emphasis was placed on recent publications to reflect the rapidly evolving evidence base, while earlier studies were retained where they provided foundational information on contaminant occurrence, toxicokinetic mechanisms, analytical methodology, or regulatory development.

Additional relevant publications were identified through backward and forward citation tracking of eligible articles and recent review papers, as well as through expert-informed literature searches to identify key mechanistic and regulatory publications not retrieved during the initial database searches. Because evidence identification remained iterative and was supplemented by citation tracking and targeted searches throughout manuscript development, the searches did not generate a single record set for formal deduplication or sequential title/abstract and full-text screening.

EFSA scientific opinions concerning edible insects and insect-derived novel foods, together with relevant EU legislation and guidance documents, were identified through the EFSA website and EUR-Lex and incorporated where relevant to the regulatory interpretation of chemical hazards.

### 2.3. Source Selection and Evidence Evaluation

Sources were considered for inclusion when they provided evidence relevant to contaminant occurrence, transfer, accumulation, metabolism, transformation, processing- or storage-related formation, dietary exposure, or regulatory assessment of edible insects and insect-based foods. Priority was given to peer-reviewed studies investigating the occurrence, transfer, accumulation, metabolism, transformation, or formation of chemical contaminants in edible insects or insect-derived foods, together with authoritative scientific opinions and regulatory documents relevant to food safety. Primary research articles were prioritised for evidence concerning contaminant occurrence and behaviour, whereas reviews and authoritative scientific reports were used principally to contextualise the evidence base and identify relevant primary studies. EFSA scientific opinions and EU legal acts were treated as primary sources for the regulatory analysis.

For interpretation, evidence was classified according to its direct relevance to human food safety. Studies of commercially available insect foods and products provided the most direct evidence of market occurrence. Studies of farmed insects reared under food-relevant production conditions were considered directly informative for potential food-chain contamination but not necessarily representative of the wider market. Controlled exposure studies, including experiments using deliberately contaminated substrates, were used primarily to evaluate transfer, accumulation, metabolism, and transformation mechanisms. Studies involving insects intended for animal feed and wild-collected insects or environmental and biomonitoring studies were retained where they provided relevant mechanistic or exposure-pathway information, but their findings were not interpreted as representative of contaminant occurrence in commercially produced foods intended for human consumption.

This evidence framework was applied throughout the synthesis so that market-occurrence, food-relevant production, controlled-exposure, feed-related, and environmental evidence were not treated as interchangeable. The final synthesis drew on 130 cited sources, including primary research articles, review and background publications, EFSA scientific opinions, EU legislation, and other authoritative documents. This number represents the sources incorporated into the narrative synthesis rather than records selected through a systematic screening process.

### 2.4. Evidence Synthesis

The collected literature was synthesised qualitatively by contaminant category while identifying common exposure pathways, bioaccumulation patterns, and regulatory implications across contaminant groups. Within each contaminant category, findings were interpreted according to the evidence framework described above, so that mechanistic, environmental, feed-related, production-stage, product-specific, and market-occurrence evidence were not treated as interchangeable. Because of substantial heterogeneity in insect species, developmental stages, production systems, substrates, analytical methodologies, and reported outcome measures, a quantitative meta-analysis was not considered appropriate.

The review was prepared collaboratively by the four authors. The principal thematic sections were initially divided among the authors according to subject expertise, after which all sections underwent cross-review by the remaining authors to improve consistency, completeness, and balance of interpretation before preparation of the final manuscript. Throughout the synthesis, contaminant occurrence, dietary exposure, health-risk characterisation, and regulatory compliance were treated as distinct interpretive levels. Detection or measurement of a contaminant in an insect or insect-based product establishes occurrence but does not by itself demonstrate meaningful dietary exposure or consumer risk. Dietary exposure additionally depends on consumption amount and frequency, product form, and exposure from other dietary sources, whereas risk characterisation requires integration of exposure with relevant toxicological information. Regulatory compliance was considered separately: comparison with a limit or reference value established for another food category was treated as contextual information only and was not interpreted as evidence of legal non-compliance unless that value is applicable to the insect product concerned.

## 3. Chemical Food Safety Hazards in Edible Insects

### 3.1. Heavy Metals and Metalloids (Cd, Pb, Hg, As)

Heavy metals such as cadmium (Cd), lead (Pb), mercury (Hg), and metalloids such as arsenic (As) are naturally present in environmental matrices, including soil, water, and plants, but anthropogenic activities can significantly increase their concentrations. As a result, these elements may enter insect production systems via contaminated substrates or environmental exposure.

Insects can accumulate heavy metals during feeding and development, particularly when reared on contaminated substrates, making them potential vectors of these contaminants in the food chain.

The relevance and interpretation of this pathway differ between wild-collected and farmed insects. Wild-collected insects may reflect local environmental contamination through contact with soil, vegetation, water, and atmospheric deposition, whereas farmed insects are primarily exposed through rearing substrates, feed ingredients, and the production environment. Therefore, substrate quality is a key determinant of heavy metal occurrence in commercially produced insects.

The context of the available studies should also be considered when interpreting reported concentrations. Some studies have examined insects as environmental bioindicators or as components of ecological food chains, while others have evaluated farmed species under controlled feeding conditions for food or feed safety purposes. Consequently, elevated concentrations reported in wild-collected insects or experimentally contaminated substrates should not be interpreted as directly representative of commercially produced edible insects. Table 1 summarises study-level Cd and Pb data together with the study setting, exposure or production context, concentration basis, sample or study design, and analytical method.

Recent market-oriented studies provide direct occurrence data for commercially available edible-insect products, although their sampling frames and approaches to interpretation differ substantially. Gori et al. [14] analysed 52 insect-based products marketed through European e-commerce and compared Pb and Cd concentrations with product-specific specifications established for the relevant insect novel foods, while Ni concentrations were contextualised using EFSA monitoring data. San Onofre et al. [15] analysed 31 products obtained from a single e-commerce platform, comprising 13 whole-insect products and 18 insect-based foods originating from Europe, Asia, and the Americas. Holowaty et al. [16] analysed 12 whole-insect products from the French online market and interpreted As, Cd, and Pb concentrations in relation to FAO/WHO provisional tolerable daily intake values. These studies confirm that potentially toxic elements can occur in marketed products, but their limited and heterogeneous sampling frames and different comparison approaches do not support a market-wide conclusion on regulatory compliance. Controlled feeding studies and previous reviews further demonstrate that metal transfer and accumulation depend on the contaminant, substrate, insect species, and developmental stage [17,18,19,20,21].

**Table 1:** Study-level evidence on cadmium and lead concentrations reported in selected insect studies, stratified by evidence category and exposure context.

Species, Stage or Product	Study Context	Study and Analytical Design	Cd (mg/kg)	Pb (mg/kg)	Ref.
*Hermetia illucens* Larvae	Switzerland; controlled exposure; nominal feed concentrations (μg/g): Cd 0, 2, 10, 50; Pb 0, 5, 25, 125	3 replicates per treatment; 200 larvae initially per replicate; dw; HR-ICP-MS	0.2 ± 0.02; 7.0 ± 0.3; 32.5 ± 0.6; 170.5 ± 8.5	n.d.; 3.8 ± 0.4; 22.8 ± 1.6; 141.7 ± 17.2	Diener et al. [17]
*Hermetia illucens* Adults	Switzerland; controlled exposure; same exposure cohorts as larvae above	3 replicates per treatment; dw; HR-ICP-MS	n.d.; 0.6 ± 0.04; 1.9 ± 0.2; 7.8 ± 0.4	n.d.; n.d.; 5.9 ± 0.57; 17.3 ± 1.36	Diener et al. [17]
*Hermetia illucens* Young larvae (pre-trial) and 5th–6th instar larvae	Germany; feed-related controlled rearing; trial substrates: mixture of middlings (control), DDGS (protein), dried sugar beet pulp (fibre)	Young larvae: 1 sample; trial groups: 7 replicates per substrate; DM; VDLUFA 10.8.1.2	0.36 (young); 0.47 ± 0.06; 0.60 ± 0.05; 2.24 ± 0.31	0.77 (young); 2.68 ± 0.26; 0.55 ± 0.19; 1.16 ± 0.14	Tschirner and Simon [21]
*Musca domestica* Dried larvae	UK, Ghana, Mali, China; feed-related production survey; locally used low- or zero-value waste substrates	5 production samples: UK *n* = 1, Ghana *n* = 1, Mali *n* = 1, China *n* = 2; MD1–MD5 not linked to individual countries; dried samples; moisture basis otherwise NR; ICP-MS	0.334; 0.625; 0.348; 0.711; 0.723	NR	Charlton et al. [22]
*Calliphora vomitoria* Dried larvae	UK; feed-related production survey; local waste substrates	2 production samples; dried samples; moisture basis otherwise NR; ICP-MS	0.020; 0.018	NR	Charlton et al. [22]
*Chrysomya* spp. Dried larvae	Ghana; feed-related production survey; local waste substrate	1 production sample; dried samples; moisture basis otherwise NR; ICP-MS	0.370	NR	Charlton et al. [22]
*Hermetia illucens* Dried larvae	Ghana; feed-related production survey; local waste substrate	1 production sample; dried sample; moisture basis otherwise NR; ICP-MS	0.120	NR	Charlton et al. [22]
*Tenebrio molitor* Last-instar larvae	Italy; controlled rearing; five experimental diets: 100% organic wheat flour; 100% organic wheatmeal; wheatmeal with 25%, 50% or 75% organic olive pomace	4000 first-instar larvae per experimental diet; 3 replicates per diet; ww; GF-AAS	0.011 ± 0.001; 0.016 ± 0.002; 0.011 ± 0.001; 0.008 ± 0.001; 0.011 ± 0.002	0.066 ± 0.008; 0.079 ± 0.005; 0.066 ± 0.004; 0.063 ± 0.005; 0.073 ± 0.003	Truzzi et al. [23]
*Zophobas morio* Last/penultimate larval instar	Czech Republic; controlled feed comparison; wheat bran; oat bran; soy flour	3 feed groups; 3 repeated measurements; biological sample size NR; DM; handheld ED-XRF	230 ± 9; 163 ± 22; 147 ± 28	<LOD; <LOD; <LOD	Mlček et al. [24]
*Tenebrio molitor* Last/penultimate larval instar	Czech Republic; controlled feed comparison; wheat bran; oat bran; soy flour	3 feed groups; 3 repeated measurements; biological sample size NR; DM; handheld ED-XRF	183 ± 23; 157 ± 18; 186 ± 27	<LOD; <LOD; <LOD	Mlček et al. [24]
*Zonocerus variegatus* Insect sample; stage NR	Nigeria (Dutsin-Ma, Katsina State); locally collected from 4 sites	3 samples per species; triplicate determinations; dry sample; AAS	7.11 ± 0.89	0.70 ± 0.58	Abdulahi et al. [25]
*Macrotermes bellicosus* Insect sample; stage NR	Nigeria (Dutsin-Ma, Katsina State); locally collected from 4 sites	3 samples per species; triplicate determinations; dry sample; AAS	1.74 ± 0.00	0.23 ± 0.06	Abdulahi et al. [25]
*Schistocerca gregaria* Insect sample; stage NR	Nigeria (Dutsin-Ma, Katsina State); locally collected from 4 sites	3 samples per species; triplicate determinations; dry sample; AAS	3.51 ± 1.62	0.04 ± 0.08	Abdulahi et al. [25]

Note: Concentrations are presented on the basis reported in the original studies. Where multiple treatments or samples were analysed within a study, individual values are retained in the same cell rather than collapsed into a species-level minimum–maximum range; where treatment order is specified under “Study context”, values are presented in the corresponding order. For Ref. [22], the five *Musca domestica* values correspond to samples MD1–MD5; these sample codes were not linked to individual countries in the source. Values are not pooled across studies. Values reported with ± represent mean ± SE in Ref. [17] and mean ± SD in Refs. [21,23,24,25]; values in Ref. [22] represent individual production samples. Controlled-exposure, feed-related, and locally collected studies are included to characterise transfer and accumulation potential and should not be interpreted as representative occurrence data for commercially marketed edible-insect foods. Values from Ref. [22] were converted from µg/kg to mg/kg. Hg and As results are discussed in the accompanying text rather than tabulated; Ref. [26] is therefore not included as a data row in this Cd/Pb table. NR, not reported for the specified analyte or parameter; n.d., not detected as reported by the original study; LOD, limit of detection; in Ref. [24], Pb was reported as <LOD, but the numerical LOD was not provided; DM, dry matter; dw, dry weight; ww, wet weight; DDGS, dried distillers’ grains with solubles; HR-ICP-MS, high-resolution inductively coupled plasma mass spectrometry; ICP-MS, inductively coupled plasma mass spectrometry; GF-AAS, graphite furnace atomic absorption spectrometry; ED-XRF, energy-dispersive X-ray fluorescence spectrometry; AAS, atomic absorption spectrometry. No EU novel-food authorisation status is inferred from taxonomic identity. In Ref. [25], the source spells the locust genus “Schisocerca”; the accepted spelling *Schistocerca gregaria* is used here.

Concentrations of Cd reported in the literature show substantial variability across insect species, life stages, evidence categories, and exposure scenarios (Table 1). Because the available evidence includes food-relevant rearing studies, feed-related investigations, deliberately contaminated-substrate experiments, and wild or environmentally exposed insects, the reported concentrations should not be interpreted as a single comparable occurrence range. Some of the highest concentrations reported in Table 1 originate from controlled high-exposure experiments or non-market environmental samples and therefore indicate accumulation potential rather than representative occurrence in commercially marketed edible-insect foods [17,24,25]. This variability reflects differences in substrate composition, environmental contamination, and production systems rather than inherent species characteristics alone [20,23,27,28].

A similar pattern is observed for Pb. Reported concentrations vary considerably across species, life stages, evidence categories, and exposure scenarios (Table 1). Regulation (EU) 2023/915 establishes Pb maximum levels for specified food categories [29]; however, values established for other food categories should not be assumed to apply directly to edible insects. Direct comparison of the reported Pb concentrations is further limited by differences in insect origin, concentration basis, and exposure conditions. Environmental contamination appears to be an important determinant of Pb levels, with higher concentrations frequently associated with insects collected from polluted environments [30]. Higher concentrations reported in wild-collected insects or controlled exposure studies are useful for identifying transfer and accumulation potential under elevated exposure conditions but should not be interpreted as representative market-occurrence data for commercially produced edible insects.

In contrast, available data for Hg and As are more limited than those for Cd and Pb and are therefore more appropriately considered narratively. In a food-relevant controlled rearing study of *Tenebrio molitor*, Hg concentrations of 0.00012–0.00049 mg/kg and As concentrations of 0.021–0.023 mg/kg were reported on a wet-weight basis across five feeding treatments [23]. Controlled substrate studies also demonstrate that uptake of these elements can increase under manipulated exposure conditions [19]. By contrast, Zhang et al. reported a mean total Hg concentration of 0.167 mg/kg in grasshoppers sampled from an industrially polluted area in Huludao, China [26]; this represents environmental biomonitoring evidence rather than occurrence in commercially produced edible insects. Interpretation of As is further constrained by the frequent lack of speciation between inorganic and organic forms, which limits assessment of toxicological relevance. Another important consideration is that total elemental concentration does not necessarily reflect the fraction that becomes available for gastrointestinal absorption after consumption. Recent in vitro digestion studies indicate that the bioaccessibility of several metals differs considerably among insect species and elements, suggesting that exposure estimates based solely on total concentrations may either overestimate or underestimate internal exposure. Consequently, bioaccessibility data should increasingly complement occurrence studies in future dietary risk assessments [31].

Overall, the available evidence shows that elemental concentrations in insects are strongly influenced by species, developmental stage, substrate composition, production conditions, and environmental exposure. However, evidence from controlled high-exposure or environmental studies should be clearly distinguished from occurrence data for commercially marketed foods. Comparisons with limits established for other food categories may provide contextual information but, unless a limit is legally applicable to the insect product concerned, they do not establish regulatory non-compliance or consumer risk. Dietary exposure and risk characterisation additionally require consideration of consumption, bioaccessibility or speciation where relevant, and appropriate toxicological reference values.

### 3.2. Mycotoxins

Insects may act as carriers of fungal spores and thereby contribute to the dissemination of mycotoxigenic fungi. Mycotoxins produced by field or storage fungi, including aflatoxins (Afs), ochratoxin A (OTA), zearalenone (ZEN), and fumonisins, may also be detected in insect biomass [32]. Stored-grain insect pests such as the Angoumois grain moth (*Sitotroga cerealella*), red flour beetle (*Tribolium castaneum*), and lesser grain borer (*Rhyzopertha dominica*) have been shown to carry spores of mycotoxigenic *Aspergillus*, *Fusarium*, and *Penicillium* species and to contribute to fungal contamination of stored maize, wheat, and rice [33,34]. Although these studies concern storage pests rather than farmed edible insects, they illustrate the potential role of insects in the dissemination of mycotoxigenic fungi.

Edible insects may be exposed to mycotoxigenic fungi or mycotoxins through contaminated rearing substrates and feed materials, including brewer’s yeast, wheat flour, ground oats, grain bran, and poultry feed, or during processing and storage [35]. The influence of storage practices is illustrated by a study from Zimbabwe, in which AfB_1_ was detected at 0.5–0.6 µg/kg in edible stink bugs (*Encosternum delegorguei*) stored in traditionally woven, dung-smeared baskets or gunny bags previously used to store cereals, whereas insects kept in clean zip-lock bags contained no detectable amount of it [36].

Mycotoxins have also been detected in insect products. In Belgium, alternariol and HT-2 toxin were detected in yellow mealworm larvae (*T. molitor*), while alternariol methyl ether was detected in adult house crickets (*A. domesticus*). ZEN was detected in black soldier fly larvae (*Hermetia illucens*), and nivalenol in migratory locusts (*L. migratoria*) [37]. Kachapulula et al. [38] likewise measured Af concentrations of 3–25 µg/kg in dried caterpillars and 16–37 µg/kg in termites collected from Zambian markets.

The fate of mycotoxins in insects appears to depend on the insect species, the toxin involved, the exposure concentration, and the composition of the rearing substrate. Coleoptera species have shown a high capacity to excrete mycotoxins, whereas the black soldier fly (*H. illucens*) showed a lower excretion capacity for OTA, ZEN, and deoxynivalenol (DON) [35]. Interactions among co-occurring mycotoxins may also modify their toxicokinetic behaviour [35,39].

Experimental studies generally indicate limited accumulation of parent mycotoxins in insects, although species-dependent differences have been reported. Camenzuli et al. [40] investigated AfB_1_, DON, OTA, and ZEN in lesser mealworm (*Alphitobius diaperinus*) and black soldier fly larvae. The results did not indicate substantial accumulation of these parent mycotoxins, although low concentrations of AfB_1_ and ZEN metabolites were detected.

Species-specific variation was also observed for ZEN. Bily et al. [41] did not detect ZEN in European corn borer (*Ostrinia nubilalis*) larvae, whereas approximately 650 µg/kg was measured in corn earworm (*Helicoverpa zeae*) larvae after four days of exposure. ZEN concentrations subsequently declined and were no longer measurable in pupae or adults, suggesting that the larvae may be capable of degrading ZEN. Similarly, AfB_1_ did not accumulate substantially in black soldier fly or yellow mealworm larvae, although AfM_1_ was detected only in yellow mealworm larvae, suggesting differences in AfB_1_ metabolism between species. The authors nevertheless noted that the fate of additional AfB_1_ metabolites remains uncertain [42].

More recent evidence has extended this work beyond the measurement of parent compounds and known metabolites. Tao et al. [43] evaluated larvae of black soldier fly, housefly, and lesser mealworm reared on aflatoxin B_1_-contaminated substrates and found no bioaccumulation of aflatoxin B_1_, while the observed metabolic patterns differed among insect species. Larval extracts from houseflies showed no cytotoxic or genotoxic potential under the conditions tested, whereas interpretation for black soldier fly and lesser mealworm larvae was limited by insect–matrix effects in the applied in vitro assays. These findings illustrate that toxicological evaluation of insect biomass may require matrix-adapted testing approaches in addition to residue analysis.

Overall, current evidence suggests that edible insects generally do not substantially accumulate the parent mycotoxins investigated to date. However, mycotoxin fate varies according to insect species, toxin type, exposure conditions, and substrate composition, meaning that concentrations in insect biomass cannot be reliably predicted from contamination levels in the rearing substrate alone. Important uncertainties also remain regarding the formation, identity, and toxicological relevance of mycotoxin metabolites and other transformation products. Consequently, low concentrations of parent mycotoxins demonstrate only limited occurrence of the measured parent compounds and should not, in isolation, be interpreted as evidence of negligible dietary exposure or consumer risk, particularly where metabolite formation remains incompletely characterised. Most available toxicokinetic evidence derives from controlled exposure studies and therefore informs mycotoxin fate and transformation rather than occurrence under commercial production conditions. Further studies under food-relevant production conditions, together with occurrence data from commercially available products, are needed to characterise species-specific toxicokinetics, metabolite formation, and combined exposure under consumer-relevant scenarios. Recent developments in multi-mycotoxin analysis may support this objective by enabling the simultaneous determination of parent mycotoxins and selected metabolites in substrates, insect biomass, and residual material. Such matrix-specific analytical methods will be important for future mass-balance studies and routine monitoring of insects reared on potentially contaminated side streams [44].

### 3.3. Pesticide Residues

Pesticides, including insecticides, herbicides, and fungicides, are widely used in agricultural systems and may contaminate environmental matrices such as soil, vegetation, and water. Insects may consequently be exposed through direct contact, ingestion of contaminated plant material or water, or contact with contaminated surfaces, potentially resulting in pesticide residues in insect biomass [45].

Pesticide residues may also persist in agricultural soil and vegetation beyond the period of application. Honert et al. [46] detected complex mixtures of current-use pesticides in both matrices throughout the year, including in areas adjacent to treated fields. These environmental exposure pathways are particularly relevant to wild-collected edible insects inhabiting agricultural landscapes, where previous pesticide applications and exposure conditions may be difficult to characterise and control. In contrast, commercially farmed edible insects are generally reared under more controlled conditions, where the primary source of pesticide exposure is the rearing substrate or feed ingredients. Consequently, the traceability and quality of substrates represent key factors in minimising pesticide contamination in insect production systems.

Labu et al. [47] screened six edible insect species collected or reared in Uganda and Kenya for 374 agrochemicals and detected nine pesticide residues, including insecticides, herbicides, and fungicides. Most detected concentrations were below the Codex Alimentarius MRLs for meat and meat products used by the authors as comparison benchmarks, although several herbicide and fungicide residues exceeded these values, in some cases substantially. However, these Codex MRLs are not necessarily the legally applicable EU limits for edible insects; therefore, exceedance of these comparison values should not be interpreted as evidence of EU regulatory non-compliance or, by itself, of consumer risk.

A complementary survey of wild-harvested and farmed edible insects from Uganda and Nigeria detected 23 current-use pesticides, while most legacy organochlorine pesticides were below the limit of quantification (LOQ). Several of the highest concentrations occurred in wild-harvested insects, although the estimated dietary exposure did not indicate a health concern for the adult population under the assumptions applied [48]. These findings reinforce the distinction between exposure scenarios that are difficult to control in wild harvesting and those that may be managed more effectively through substrate traceability and controlled farming. More recent market-surveillance data indicate that pesticide residues may also occur in commercially available insect products. A Japanese survey combined a matrix-adapted analytical method with the testing of marketed edible insects, demonstrating both the feasibility of routine residue monitoring and the importance of validating methods specifically for insect matrices [49].

Controlled feeding experiments provide direct evidence that residues present in feed may be transferred to farmed insects, although the extent and chemical form of transfer are compound- and species-dependent. In Jamaican field crickets (*Gryllus assimilis*) and yellow mealworms (*T. molitor*) fed carrots containing incurred pesticide residues, parent compounds as well as conjugated or bound residues were detected in insect biomass, with differences between the two species [50]. These findings show that monitoring limited to free parent compounds may not fully characterise pesticide-related residues in farmed insects. However, such controlled feeding experiments demonstrate the potential and chemical form of substrate-to-insect transfer rather than the frequency or magnitude of pesticide residues in commercially marketed insect foods. Overall, these findings indicate that edible insects may contain pesticide residues originating from contaminated habitats or rearing substrates. Residue occurrence appears to depend strongly on the insects’ habitat, feeding sources, and rearing substrates. Wild-collected insects may be exposed to pesticide applications and contaminated vegetation under conditions that are difficult to document or control, whereas farmed insects may also acquire residues when reared on contaminated organic materials. These findings highlight the importance of controlled production systems, traceable substrates, and monitoring of environmental inputs in reducing pesticide-related contamination and potential dietary exposure. To date, relatively few studies have systematically investigated pesticide residues in edible insects produced under commercial farming conditions. Much of the available evidence originates from wild-collected insects or from surveys designed to characterise environmental contamination rather than food safety. Consequently, comparisons among studies should take differences in insect origin, production system, and exposure scenario into account. Recent advances in high-resolution analytical screening methods may further improve future monitoring by enabling the simultaneous detection of a broad range of pesticide residues and other contaminants in insect matrices [51].

Within the EU, pesticide residues in insects are subject to Regulation (EC) No 396/2005, including the default MRL provision where no specific MRL is established; the regulatory implications are discussed in Section 6.2 [13].

### 3.4. Veterinary Medicinal Product Residues in Edible Insects

In edible-insect production, veterinary medicinal products (VMP) are relevant primarily because pharmacologically active substances may occur as residues following indirect exposure rather than authorised therapeutic use. In conventional livestock production, VMP are deliberately administered within established systems for treatment, prophylaxis, withdrawal periods, and residue control. By contrast, edible insects may be exposed indirectly through contaminated substrates or other rearing inputs, although VMP use for disease control may become more relevant as production intensifies. EFSA and FAO have identified these exposure pathways as potential food-safety concerns, while subsequent reviews have emphasised the limited evidence on residue occurrence, transfer, and toxicokinetics in edible insects [52,53,54,55].

Experimental studies demonstrate that pharmacologically active substances can be transferred from contaminated substrates to insect biomass and that transfer is compound-specific. In black soldier fly larvae (*Hermetia illucens*), van Dongen et al. [56] investigated enrofloxacin, oxytetracycline, sulfamethoxazole, narasin, salinomycin, toltrazuril, and eprinomectin in experimentally spiked substrates. Transfer to larvae varied considerably among compounds, and metabolites of some substances were detected in larvae and frass, indicating that residue behaviour may involve uptake, biotransformation, and excretion. Similar findings have been reported in an experimental study on yellow mealworm larvae (*T. molitor*), where tiamulin, chloramphenicol, and erythromycin remained detectable after a 24 h fasting period, despite marked decreases in concentration [57]. These controlled exposure studies demonstrate that pharmacologically active substances may be transferred, transformed, degraded, or retained in insect-rearing systems, but they should not be interpreted as evidence of the occurrence of VMP residues in commercially marketed edible-insect foods. Direct market-occurrence data remain very limited. More recent work examined the effects and residue fate of chloramphenicol, methoprene, and narasin in yellow mealworms exposed through the rearing substrate for up to 168 h. None of the substances significantly affected larval survival or weight gain under the tested conditions, although all three were detected in exposed larvae using LC–HRMS and LC–MS/MS. Metabolite screening did not identify major metabolites considered relevant to residue monitoring, suggesting that surveillance of these substances may primarily require sensitive determination of the parent compounds. Nevertheless, the observed residue concentrations and their temporal patterns were compound-specific, reinforcing the need for substance-specific transfer and depletion data [58]. Collectively, these studies show that residue depletion cannot be assumed to be rapid or complete and highlight the need for insect-specific data on uptake, metabolism, elimination, and pre-harvest interventions.

Black soldier fly larvae and their associated microbiota may also accelerate the degradation of certain pharmaceutical compounds in contaminated substrates. Substantial reductions have been reported for tetracycline, while shorter half-lives were observed for carbamazepine, roxithromycin, and trimethoprim in substrates containing larvae, without detectable accumulation of these compounds in larval biomass [52,55]. Experimental studies have also reported enhanced degradation of lincomycin and oxytetracycline in black soldier fly larval systems [59,60]. However, this pattern is compound-specific and cannot be generalised across VMP. Possible biodegradation products have been identified in some experiments, while their identity and toxicological relevance remain incompletely characterised. Reductions in parent-compound concentrations should therefore not be interpreted as evidence of complete elimination without mass-balance studies covering the substrate, larvae, frass, metabolites, and other transformation products.

Antimicrobial resistance represents an additional concern. Transferable antimicrobial resistance genes have been detected in bacteria associated with edible insects, with possible sources including contaminated substrates, water, and rearing environments [54]. This issue may be particularly relevant where substrates contain antimicrobial residues or materials of animal origin. Antimicrobial residues and resistance determinants should therefore be evaluated together, as they may arise from related production and exposure pathways [52,55].

Analytical monitoring of VMP residues in edible insects is technically feasible. De Paepe et al. [37] developed and validated a multi-residue screening method for mealworm, grasshopper, house cricket, and black soldier fly matrices, covering 29 veterinary drugs alongside pesticides and mycotoxins. Its application to commercial samples demonstrated the feasibility of broad residue surveillance in insect-derived foods. Together with recent controlled feeding studies using LC–MS/MS and LC–HRMS, this evidence indicates increasing analytical readiness for residue monitoring, while also showing that method suitability cannot simply be assumed from validation in conventional animal-derived matrices [57,58].

The current EU regulatory framework is not fully aligned with this risk profile. Regulation (EU) 2019/6 [61] governs the authorisation and use of veterinary medicinal products, while Regulation (EC) No 470/2009 [62] and Commission Regulation (EU) No 37/2010 [63] establish the framework for classifying pharmacologically active substances and setting MRLs in foodstuffs of animal origin. Authorisation of VMP for food-producing animals is target-species based, but edible-insect species are not explicitly included as authorised target species within this system [64]. Thus, because no veterinary medicinal products are currently authorised for use in food-producing insects, insect-specific data on residue depletion, withdrawal periods, target tissues, and interpretation of residue findings have not been generated within the existing authorisation framework. Consequently, commercial edible-insect production does not currently have a dedicated framework for authorising VMP use and assessing the resulting residues.

Although routine therapeutic use of VMP in edible-insect farming is not established, disease-control needs may become more important as production systems intensify. Infectious diseases are already recognised as constraints across insect species and production scales [65]. At the same time, authorised treatment options and insect-specific information on effective dosage, metabolism, residue depletion, and withdrawal periods remain largely unavailable [64,66].

Overall, veterinary drug residues represent an emerging chemical safety concern in edible insects. Their relevance arises from potential substrate-mediated exposure, possible future disease-control use, compound-specific transfer and metabolism, and the lack of insect-specific residue-assessment tools.

### 3.5. Persistent Organic Pollutants and Emerging Contaminants

Persistent organic pollutants (POPs), including polychlorinated dibenzo-p-dioxins (PCDDs), polychlorinated dibenzofurans (PCDFs), and polychlorinated biphenyls (PCBs), are lipophilic environmental contaminants capable of persisting, bioaccumulating, and biomagnifying within food chains. Due to their toxicological relevance, maximum levels have been established in the EU for several food and feed categories under separate regulatory instruments [29,67]. However, their occurrence, transfer, and behaviour in edible insects remain poorly characterised [53,68].

The rearing substrate is considered an important potential source of contamination because insects may assimilate environmental contaminants present in feed materials, organic side streams, or food-industry by-products. The likelihood and extent of transfer into insect biomass may vary according to the contaminant, insect species, substrate composition, and production conditions [18,27]. Although EU legislation restricts the use of certain waste streams in insect production, contamination originating from authorised feed materials or environmental sources cannot be entirely excluded [53,69]. In a screening study that included black soldier fly larvae (*Hermetia illucens*), dioxins and PCBs were below the detection limit; polycyclic aromatic hydrocarbons (PAHs) were also analysed. However, contaminant concentrations in the corresponding rearing substrates were not reported, preventing assessment of substrate-to-insect transfer or bioaccumulation [69]. Recent controlled evidence further illustrates the importance of analysing both substrates and insect biomass. Papin et al. [70] compared black soldier fly larvae reared on EU-authorised food by-products and surplus materials with larvae reared on unauthorised biowaste streams. POPs, pesticides, and trace elements were detected in parts of the production system, whereas per- and polyfluoroalkyl substances (PFAS) and mycotoxins were not detected in the investigated substrates. Transfer and accumulation patterns differed among contaminant classes, demonstrating that the chemical safety of insect biomass cannot be inferred solely from the legal classification of the substrate and must be verified analytically. As a controlled substrate study, however, these findings primarily inform contaminant transfer and the influence of substrate type rather than occurrence in commercially marketed insect foods. Although dioxins and PCBs have occasionally been detected in insects, the available studies remain too limited and heterogeneous to establish consistent patterns across insect species, substrates, and production systems [68]. These uncertainties highlight the need for controlled transfer studies and systematic monitoring of both rearing substrates and insect biomass.

Beyond dioxins and PCBs, increasing scientific attention has been directed towards other persistent contaminants, including PFAS and brominated flame retardants (BFRs). PFAS represent a large class of synthetic fluorinated compounds used in numerous industrial and consumer applications because of their water- and grease-repellent properties. Many PFAS are highly persistent and widely distributed in the environment, and exposure to some compounds has been associated with adverse health effects, including immunotoxic, reproductive, and teratogenic potential [71,72].

A recent study reported the occurrence of PFAS in edible insect products. In a survey of 34 commercially available products derived from house cricket (*A. domesticus*), migratory locust (*Locusta migratoria*), and yellow mealworm (*T. molitor*), PFAS were detected in all samples, with differences in concentrations and congener profiles among species [73]. This study provides direct market-occurrence evidence for commercially available products derived from EU-authorised edible-insect species. However, because information on rearing substrates and production conditions was incomplete, the observed differences among products cannot be attributed unequivocally to insect species, substrate, or production system.

BFRs, including polybrominated diphenyl ethers (PBDEs), comprise compounds with persistent and bioaccumulative properties that have been detected in environmental matrices and food chains. Despite increasing regulatory restrictions, some remain relevant because of their continued presence in products, waste streams, and the environment [74,75]. Evidence concerning their occurrence and behaviour in edible-insect production systems remains very limited. In one experimental study, hexabromocyclododecane (HBCD) did not bioaccumulate in yellow mealworm (*T. molitor*) tissues following exposure through HBCD-containing polystyrene, although these experimental conditions were not representative of conventional edible-insect production [76].

Overall, the evidence base differs markedly among persistent contaminant groups. Market-occurrence data are now available for PFAS in several EU-authorised edible-insect species, whereas controlled information on contamination sources, substrate-to-insect transfer, bioaccumulation, and elimination remains scarce for PFAS, dioxins, PCBs, and BFRs.

### 3.6. Processing and Storage-Related Chemical Hazards

Technological processing and storage may introduce or promote a distinct group of chemical hazards in edible insects and insect-based foods, even when contaminants originating from the rearing substrate are excluded. The available insect-specific evidence is concentrated in three main areas: heat-induced process contaminants, oxidative deterioration during storage, and biogenic amines associated primarily with fermentation. Key characteristics of the studies addressing heat-induced process contaminants are summarised in Table 2. In contrast, primary insect-specific data remain limited for other contaminant classes of potential relevance, including PAHs, nitrosamines, packaging migrants, and mineral-oil hydrocarbons.

#### 3.6.1. Heat-Induced Process Contaminants

Current insect-specific evidence on heat-induced process contaminants has focused mainly on acrylamide and furan derivatives. González-Gómez et al. [77] developed and validated an HPLC-QqQ-MS/MS method for the simultaneous determination of acrylamide, furfural, 5-methylfurfural, and 5-hydroxymethylfurfural (HMF) in insect-based foods, including bars, crackers, and flours. Application of the method to 11 commercial insect-based products showed considerable variation among product categories. Acrylamide was below the limit of quantification in several products but was detected in baked cracker-type products, suggesting that thermal processing and product formulation, rather than the insect ingredient itself, are the primary determinants of acrylamide formation. Furan derivatives, particularly HMF, were detected mainly in bar-type products, although the insect-based bars generally contained lower HMF concentrations than the conventional cereal bars included for comparison.

González et al. [78] quantified HMF in cereal and insect bars and reported concentrations ranging from 336 to 962 mg/kg. High concentrations were found in all analysed samples, indicating that both cereal- and insect-based bars may contain substantial amounts of this process contaminant. However, the measured concentrations cannot be attributed to the insect ingredient alone, because these products contain multiple components, including dried fruits, chocolate, nuts, honey, and caramel, some of which may already contain HMF, while additional formation may occur during processing and storage.

More direct evidence of process-contaminant formation was provided by Hradecká et al. [79], who investigated salty crackers enriched with cricket powder. Acrylamide concentrations increased significantly with both cricket-powder incorporation and prolonged baking, particularly in crackers prepared with white wheat flour. The study also examined 2-MCPD and 3-MCPD fatty acid esters, as well as glycidyl fatty acid esters. The concentrations of 2-MCPD esters remained below the limit of quantification, while glycidyl ester concentrations, expressed as glycidol, did not increase with either cricket-powder addition or prolonged baking. In contrast, 3-MCPD ester concentrations increased under more intensive baking conditions, particularly after 45 min. These findings indicate that cricket-powder incorporation may increase the availability of acrylamide precursors, particularly free asparagine, while formulation and baking conditions together shape the broader process-contaminant profile of baked insect-based foods.

Fermentation has also been investigated as a mitigation strategy for acrylamide formation. Bartkienė et al. [80] showed that fermentation of cricket flour with *Lactiplantibacillus plantarum* No. 122 reduced acrylamide concentrations in wheat bread by 33.2% and 31.7% at inclusion levels of 10% and 20%, respectively, compared with breads containing corresponding amounts of untreated cricket flour. No significant reduction was observed at 30% inclusion, and most cricket-enriched breads still contained higher acrylamide concentrations than the wheat-bread control. The observed differences were linked to the availability of acrylamide precursors in cricket flour and to interactions between product formulation and baking-induced reactions. These findings suggest that fermentation can contribute to acrylamide mitigation in insect-enriched bakery products, but its effectiveness depends on the formulation and processing conditions.

#### 3.6.2. Lipid Oxidation During Storage

Lipid oxidation is one of the most consistently reported storage-related quality and stability concerns in edible insect products, particularly in powders and flours. Marzoli et al. [81] evaluated the microbiological and chemical stability of *A. domesticus* powders produced by oven drying at 80 or 120 °C or by freeze-drying and stored at room temperature for 12 months. Lipid oxidation was assessed using peroxide value and the concentrations of the secondary oxidation products hexanal and pentanal. Although aldehyde concentrations varied during storage rather than increasing continuously, freeze-dried powders generally contained lower concentrations of hexanal and pentanal than oven-dried powders. The authors therefore suggested that the drying method influenced the oxidative status of the powders during storage, with oven-dried products showing greater aldehyde formation under the conditions examined. These findings indicate that post-harvest processing may affect both the initial oxidative status of insect powders and the subsequent development of oxidation-related deterioration during storage.

The importance of storage conditions was further supported by Orkusz et al. [82], who evaluated *A. domesticus* flour after 12 months of storage at −18, 4, or 20 °C, with light exposure additionally assessed at 20 °C. The lowest peroxide value was observed under frozen storage, whereas the highest acid, peroxide, and p-anisidine values occurred at 20 °C in the presence of light. Storage at room temperature was also associated with a reduction in polyunsaturated fatty acids, while storage at −18 or 4 °C largely preserved the fatty acid profile. These findings indicate that lower storage temperatures limit oxidative deterioration, whereas light exposure can further accelerate lipid deterioration under room-temperature conditions.

Earlier work by Kamau et al. [83] supports the same conclusion. In semi-processed adult *A. domesticus* meal stored for six months under ambient or refrigerated conditions in different packaging materials, iodine values decreased, whereas peroxide and p-anisidine values increased over time. Oxidative deterioration was more pronounced under ambient storage and was also influenced by packaging, with woven polypropylene providing the least protection and rigid propylene containers the greatest. Total viable counts and yeast and mould counts also increased during storage, particularly under ambient conditions, and *Aspergillus*, *Alternaria*, and *Penicillium* spp. were isolated. These findings indicate that storage temperature, packaging, and storage duration affect both the oxidative and microbiological stability of house cricket meals.

Additional evidence is available for processed snack-type products. Gan et al. [84] evaluated lipid deterioration in deep-fried *Gryllus bimaculatus* during 150 days of storage and found that free fatty acid content, peroxide value, and thiobarbituric acid-reactive substances increased over time. Samples treated with antioxidants and packaged under vacuum with nitrogen generally showed lower values than the non-vacuum control, with butylated hydroxytoluene providing the greatest protection among the antioxidants tested. These findings indicate that antioxidant treatment and reduced-oxygen packaging can limit, but not prevent, lipid deterioration in deep-fried cricket products during storage. Similarly, Balzan et al. [85] found variable and, in some cases, pronounced lipid oxidation in commercially available shelf-stable insect products, indicating that oxidative deterioration also occurs in marketed products and is not confined to controlled storage studies. The marked variability among products further suggests that processing history, formulation, packaging, and storage conditions may all contribute to the oxidative status of marketed insect products.

#### 3.6.3. Biogenic Amines and Fermentation-Related Risks

Biogenic amines represent a potential processing-related concern in fermented insect ingredients. Bartkienė et al. [80] showed that fermentation of cricket flour with *Lactiplantibacillus plantarum* No. 122 altered the biogenic amine profile. Total biogenic amine concentrations decreased after 24 and 48 h of fermentation, and histamine was not detected in either untreated or fermented samples. However, tyramine increased markedly, whereas tryptamine, cadaverine, spermidine, and spermine generally decreased. These findings show that a reduction in total biogenic amines does not necessarily indicate a uniform reduction in individual compounds. Although fermentation reduced acrylamide formation in some of the corresponding bread formulations, it simultaneously promoted tyramine formation in the cricket flour. Therefore, the effects of starter cultures and fermentation conditions on individual biogenic amines should be considered when developing fermented insect ingredients.

Overall, the available evidence shows that processing and storage conditions can substantially influence the chemical profile of insect-based foods. Heat treatment may promote the formation of acrylamide and furan derivatives, while storage conditions affect lipid oxidation and fermentation may alter biogenic amine profiles. However, most studies remain product- and process-specific, and direct evidence linking these changes to consumer risk is still limited. Further work is needed to establish appropriate contaminant-specific reference values or interpretative benchmarks, identify the most influential processing variables, and evaluate a broader range of commercially relevant insect products.

## 4. Other Food Safety Hazards Relevant to the Chemical-Safety Context

Biological, physical, and allergenic hazards fall outside the primary scope of this review. They are therefore summarised only briefly, with emphasis on aspects that intersect with chemical safety or are necessary to place chemical hazards within the broader food-safety context of edible insects.

### 4.1. Biological Hazards in Edible Insects and Insect-Based Foods: Relevance to Chemical Safety

Biological hazards are not the primary focus of the present review; however, they remain relevant where microbiological contamination may give rise to chemically mediated hazards. In edible insects and insect-based foods, a recurrent concern is the persistence of spore-forming bacteria, particularly members of the *Bacillus cereus* group. Thermal processing can substantially reduce vegetative microbial populations, whereas bacterial endospores are more resistant and may persist in processed products [86,87,88]. Accordingly, effective control of vegetative bacteria does not necessarily eliminate the risk associated with surviving spore-formers.

This is particularly relevant from a chemical safety perspective because some spore-forming bacteria can produce toxins that remain present after viable bacterial cells are no longer detectable. Cereulide, the heat-stable emetic toxin produced by certain strains of the *B. cereus* group, is an important example. Pal et al. [88] identified 34 *B. cereus* group isolates in processed edible insect products, including three biovar *Emeticus* isolates from mealworms and mole crickets carrying the cereulide synthetase genes. Although cereulide itself was not measured, these findings confirm the presence of strains with the genetic potential to produce the toxin. Toxin formation would nevertheless depend on conditions permitting spore germination and bacterial growth. In such cases, microbiological monitoring may need to be complemented by direct chemical analysis of the toxin.

Parasitological hazards have also been reported in insects from poorly controlled small-scale production systems [89]. Although pharmacological control of certain insect parasites has been discussed, evidence of routine treatment or associated residues in edible-insect production remains lacking; this issue is therefore relevant to the present review primarily through its potential interface with veterinary–medicinal-product residues.

Overall, biological hazards are relevant to the present review primarily where they give rise to chemically mediated hazards, particularly microbial toxins.

### 4.2. Physical Hazards

Physical hazards associated with edible insects include rigid chitinous structures, legs and wings, coarse exoskeletal fragments, and extraneous materials introduced during production or processing. Although systematic evidence is limited, case reports and experimental observations indicate that intact insect structures may present mechanical risks, particularly when whole insects are inadequately processed [53,90]. Foreign materials such as metal or plastic fragments and substrate residues are generally managed through standard food-safety practices, including sorting, sieving, filtration, and metal detection, while milling and removal of rigid body parts may further reduce mechanical risk [91]. Given that these hazards do not directly concern chemical safety, they are not discussed further in this review.

### 4.3. Allergenicity of Insect Proteins

Edible insects contain proteins with allergenic potential and may cause both primary sensitisation and cross-reactive allergic responses. Tropomyosin and arginine kinase are among the best-characterised arthropod pan-allergens, and structural similarities with allergens from crustaceans, molluscs, and mites may result in clinically relevant cross-reactivity [92].

Primary allergic reactions, including anaphylaxis, have also been reported following consumption of edible insects, although population-level epidemiological data remain limited [93,94,95]. Processing may modify allergenicity, but its effects depend on the insect species, allergen, and treatment conditions. Thermal processing may reduce the immunoreactivity of some proteins, whereas several allergens, including tropomyosin, may remain comparatively heat stable. Enzymatic hydrolysis and other processing technologies have also been investigated, but no method has been shown to reliably eliminate allergenic potential from insect-derived foods [96,97,98,99,100,101,102,103,104,105].

Within the EU novel-food framework, allergenicity is assessed as a distinct safety consideration, particularly because of potential cross-reactivity with crustaceans and house dust mites. Although allergenicity lies outside the chemical-safety focus of the present review, it is retained here briefly to place chemical hazards within the broader safety assessment of insect-derived novel foods.

## 5. Cross-Cutting Exposure Pathways, Bioaccumulation Patterns, and Implications for Risk Assessment

**Figure 1:**
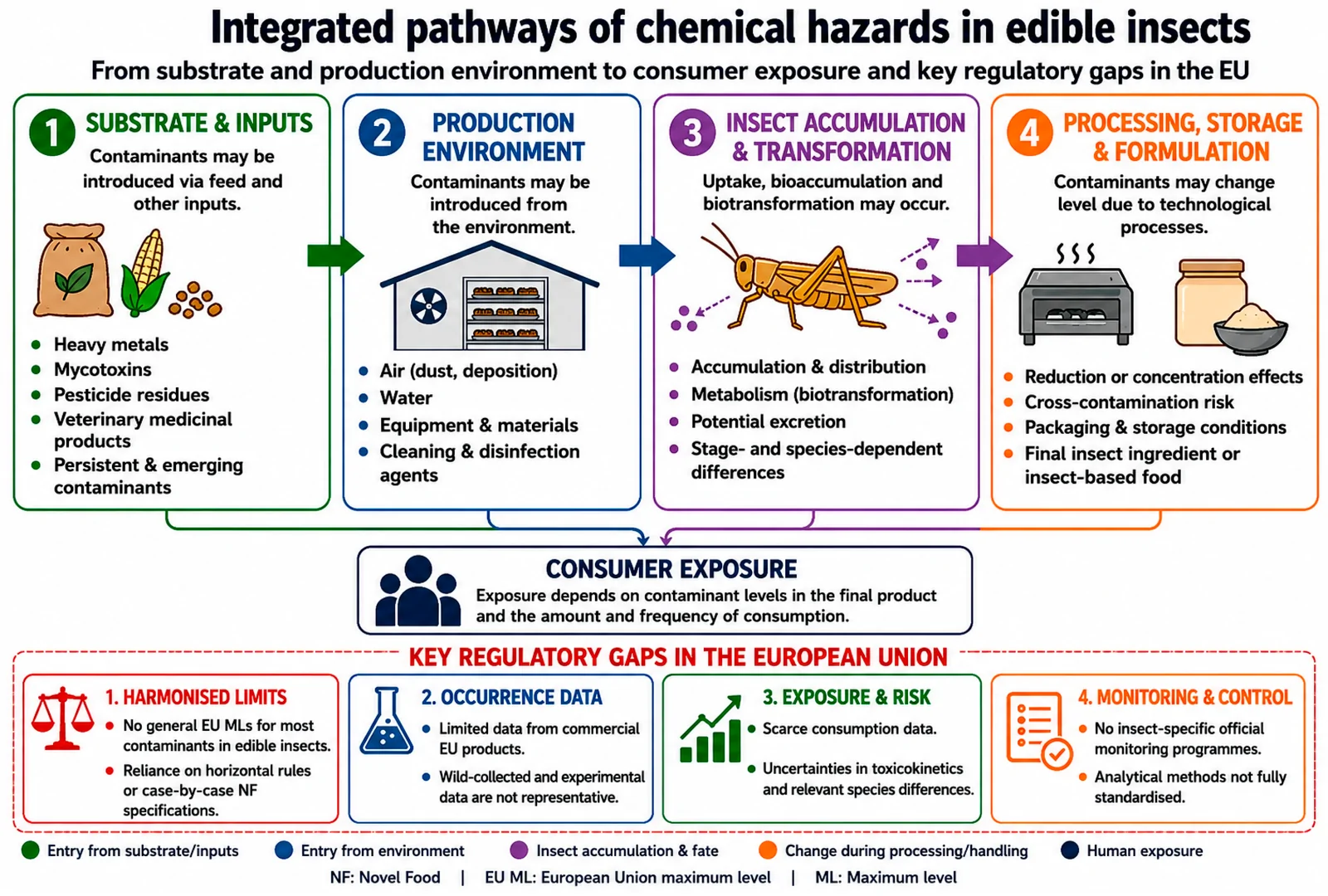
Integrated pathways of chemical hazards from rearing substrate and production environment through insect uptake, processing, and storage to final food and consumer exposure, together with key cross-cutting evidence and regulatory limitations in the European Union.

Across the contaminant groups reviewed, chemical safety is shaped by interconnected processes extending from contamination of the rearing substrate or production environment through insect uptake, metabolism, processing, storage, and final consumption. Figure 1 integrates these processes within a full-chain framework. Their relative importance varies according to the contaminant, insect species, life stage, production system, and processing and storage conditions, which limits direct extrapolation among studies and products.

### 5.1. Integrated Exposure Pathways from Substrate to Consumer

Across the contaminant groups discussed above, a common substrate- or environment-to-insect-to-food pathway emerges. Contaminants present in feed materials or the surrounding environment may be transferred to insect biomass and subsequently occur in the final food product [53,68]. Substrate quality is therefore a key determinant of contamination during the rearing phase, particularly for heavy metals, pesticide residues, mycotoxins, and veterinary drug residues, for which transfer from rearing substrates or environmental matrices has been demonstrated or considered plausible [18,56,69].

However, transfer is neither uniform nor necessarily proportional to substrate contamination. Its extent varies according to the contaminant, insect species, life stage, rearing conditions, and production system. Differences in feeding behaviour, physiology, metabolism, and excretion may influence both uptake and the concentrations ultimately detected in insect biomass. Edible insects should therefore not be treated as a uniform category from a chemical safety perspective.

Chemical hazards may also arise or be modified after harvesting. Processing and storage can change existing contaminant concentrations, generate transformation products, or introduce newly formed hazards. Thus, the chemical profile of the final product reflects both pre-harvest contamination and post-harvest transformation or formation processes.

The pathway from substrate to consumer is consequently not a simple process of contaminant transfer. Hazards may enter during rearing, undergo uptake or transformation within the insect, and be further modified or generated during processing and storage. Final consumer exposure reflects the combined influence of contamination sources, insect-specific processes, production and storage conditions, product formulation, and consumption patterns.

### 5.2. Bioaccumulation, Metabolism, and Transformation

Transfer into insect biomass does not necessarily result in simple or proportional accumulation. In several experimental systems, insects have been shown to metabolise, transform, or excrete certain contaminants, particularly selected mycotoxins and VMP residues [40,42,56]. These processes may substantially reduce the concentrations of parent compounds detected in insect tissues.

Such reductions do not necessarily indicate a corresponding decrease in toxicological risk. Biotransformation may generate metabolites or degradation products whose identity, occurrence, and toxicological properties remain insufficiently characterised. Some may retain biological activity or exhibit toxicological profiles that differ from those of the parent compounds.

Mass-balance studies further illustrate this uncertainty. In some experimental systems, part of the administered contaminant cannot be fully accounted for in insect biomass, residual substrate, or measured excreta. These unrecovered fractions may be associated with unidentified transformation products, binding to components of the insect matrix, incomplete analytical recovery, or other unmeasured losses. The absence or low concentration of a parent compound in insect tissue should therefore not be interpreted automatically as evidence of complete elimination or negligible toxicological relevance.

The relative importance of accumulation, transformation, and excretion varies among insect species and contaminants. Concentrations in insect biomass therefore cannot be reliably predicted from those in the rearing substrate alone. Species-specific physiology, life stage, and contaminant-specific chemical properties should be considered when interpreting transfer studies and comparing findings across production systems.

### 5.3. Risk Assessment Implications

These exposure and transformation patterns have direct implications for risk assessment. Analytical approaches that focus exclusively on parent compounds may not capture the full contaminant-related exposure if biologically active or toxicologically relevant metabolites are formed. At the same time, the limited availability of toxicological data for many transformation products prevents their systematic inclusion in exposure and hazard characterisation. Accordingly, the presence or concentration of a chemical hazard in insect biomass should be distinguished from dietary exposure and subsequent risk characterisation. Occurrence data describe what is present in the matrix, whereas dietary-exposure assessment additionally requires information on consumption and relevant exposure scenarios. Consumer risk can only be characterised by integrating such exposure estimates with appropriate toxicological information. Regulatory compliance represents a separate question and depends on whether a legally applicable limit or specification exists for the product concerned.

Reports of “low accumulation” should therefore be interpreted cautiously, particularly when metabolite identification is incomplete or a substantial proportion of the administered contaminants remains unaccounted. Reliable assessment requires information not only on parent-compound concentrations, but also on transformation pathways, metabolite profiles, excretion, and the toxicological relevance of the compounds formed.

Interpretation of chemical occurrence data should account for insect species, rearing conditions, product characteristics, and anticipated consumption, as these factors may substantially influence contaminant transfer and exposure. Regulatory comparisons should likewise be interpreted according to the legal applicability of the relevant limit or specification to the product concerned.

Risk assessment should therefore encompass the full production chain, including processing and storage, rather than focusing exclusively on contaminants entering through the rearing substrate. Taken together, these findings support a full-chain approach in which contaminant occurrence is interpreted in the context of substrate exposure, insect-specific fate, processing and storage, and the characteristics of the final food.

## 6. EU Regulatory Framework and Regulatory Blind Spots

The EU regulatory framework for edible insects combines case-by-case novel food authorisation with horizontal food-safety legislation applicable according to the product and contaminant concerned. Product-specific novel-food specifications may establish limits for selected chemical parameters, while applicable horizontal requirements continue to govern contaminants, pesticide residues, process contaminants, and other aspects of food safety. Regulatory uncertainties nevertheless remain where the applicability or interpretation of existing provisions is unclear, and where limited post-market occurrence and exposure data constrain monitoring and risk assessment.

### 6.1. Novel Food Authorisation and EFSA Assessments

#### 6.1.1. EU Novel Food Authorisation: EFSA Role and Data Requirements for Insects and Insect-Derived Products

In the EU, farmed insects have the status of farmed animals, and regulations applicable to livestock health and biosecurity measures for transmissible diseases also apply to farmed insects [54,106,107]. However, when insects and insect-derived products are intended to be placed on the market for human consumption, they fall within the EU NF framework and are subject to pre-market authorisation under Regulation (EU) 2015/2283, applicable as of 1 January 2018 [11]. To date, novel foods derived from four insect species have been authorised for human consumption in the EU: migratory locust (*L. migratoria*), yellow mealworm (*T. molitor* larvae), house cricket (*A. domesticus*), and lesser mealworm (*A. diaperinus* larvae) [12].

The placing of an NF on the EU market requires prior authorisation by the European Commission and inclusion in the Union list. As part of the authorisation procedure, EFSA carries out a risk assessment and issues a scientific opinion. Taking the EFSA’s opinion into account, the Commission may adopt an implementing act, after which the novel food may be marketed. In contrast to the former NF framework, under which authorisations were applicant-specific, authorisations under the current regulation are generally generic following inclusion in the Union list. This allows any food business operator to place the product on the market under the approved conditions of use and specifications, unless the authorisation is subject to temporary protection of proprietary data [11].

EFSA evaluates novel food applications by assessing whether the NF poses a risk to human health under the proposed conditions of use and whether its consumption could be nutritionally disadvantageous, particularly when intended to replace an existing food. This evaluation is conducted on the basis of a standardised set of information submitted by applicants as part of the novel food dossier, in line with EFSA guidance. EFSA first issued guidance on the scientific requirements for NF applications in 2016, which was subsequently updated in 2024 [108,109].

An NF application assessed by EFSA must include detailed information supporting the safety assessment. This includes characterisation of the NF’s identity and source, together with its relevant chemical and physical properties. The application must describe the production process in detail, from raw material handling to the final product, and identify any novel or critical processing steps. To support hazard identification and the establishment of specifications, analytical data from multiple independently produced batches are required to demonstrate compositional consistency, variability, and stability over the proposed shelf life. Based on these data, specifications are proposed that define key chemical, physical, and microbiological parameters and their limits. The application must also specify the proposed conditions of use, including target populations, intended food categories, maximum use levels, and estimates of anticipated dietary intake.

In addition, the application must include scientific evidence addressing the safety of the NF across relevant assessment areas, unless a scientifically justified omission is accepted. The submitted safety data typically follow a tiered testing strategy and include studies on absorption, distribution, metabolism, and excretion (ADME), in vitro genotoxicity testing, and, where appropriate, a 90-day subchronic oral toxicity study to identify adverse effects and establish a reference point for risk characterisation. Data addressing nutritional aspects, including antinutrients and protein quality, as well as information relevant to allergenic potential, considering possible cross-reactivity with known allergens, are also required.

On the basis of these data, EFSA concludes its assessment by integrating the toxicological, nutritional, and allergenic evidence to determine whether the NF is safe under the proposed conditions of use in relation to anticipated dietary exposure [108,109].

Applied to edible insects, this regulatory approach has resulted in several EFSA scientific opinions assessing insect-derived novel foods on a case-by-case basis, taking into account the insect species, product form, production process, and intended uses.

#### 6.1.2. Chemical and Stability Evidence Considered in EFSA Opinions on Insect-Derived Novel Foods

EFSA guidance requires novel food applications to provide information on the production process, composition, product specifications, proposed conditions of use, potential contaminants, and product stability [108,109]. This synthesis draws on 11 product-specific EFSA scientific opinions concerning insect-derived novel food applications published from 2021 through 2025 [110,111,112,113,114,115,116,117,118,119,120]. The 2015 EFSA Scientific Committee risk profile on insects as food and feed was additionally consulted for broader contextual evidence on chemical hazards and exposure pathways [53]. The 11 product-specific opinions were reviewed comparatively to identify recurring assessment approaches, differences in the evidence submitted, and limitations affecting the interpretation of chemical safety and stability data. For each opinion, available information relevant to the scope of this review was extracted from the published EFSA opinions on the novel food and product form, declarations on VMP and other pharmacologically active substances during production, batches analysed, laboratory analyses, chemical contaminants, stability, processing and intended-use studies, selected chemical safety-related specifications proposed by the applicants, and relevant EFSA discussion and conclusions. Information on analytical LODs and LOQs, corresponding controls in processing studies, and limitations identified by EFSA was retained within the relevant thematic fields where explicitly reported; information not explicitly reported was not inferred. The extracted information is presented in Supplementary Table S1, while individual assessments are cited selectively in the text as illustrative examples.

Across the reviewed opinions, applicants provided compositional and analytical data from several independently produced batches, most commonly three to five, to characterise the novel food, assess batch-to-batch variability, and support the proposed specifications. Analyses were generally conducted by accredited laboratories using methods validated for other food matrices; where in-house methods were used, detailed methodological descriptions and corresponding validation data were provided. Several insect novel foods were assessed as “whole foods” under EFSA guidance, meaning that complete identification and characterisation of all constituents was not considered feasible. The contaminants investigated varied according to the insect species, novel food form, production process, and proposed conditions of use.

Chemical contaminant analyses commonly cover heavy metals, particularly Cd, Hg and Pb, as well as As; selected mycotoxins; dioxins and dioxin-like PCBs, and pesticide residues. Depending on the application, additional analyses included PAHs, PFAS, biogenic amines, and selected processing-related contaminants. The analytes investigated, analytical methods, number of batches, and reported LODs and LOQs differed among applications. Consequently, the resulting analytical datasets were not directly comparable across all insect NFs.

Where generally applicable insect-specific MLs were not established, EFSA contextualised the reported concentrations using regulatory values or occurrence data available for other food categories. This approach was applied to heavy metals, mycotoxins, dioxins, and dioxin-like compounds, and selected processing-related contaminants. Such comparisons supported the assessment of the individual NF but did not imply that the referenced limits were legally applicable to edible insects.

The evidence used for this contextual interpretation varied among contaminant groups and applications. Mycotoxin results were frequently below the analytical LOQs; where concentrations were quantifiable, they were generally below the comparison values established for other foodstuffs. Heavy-metal results were sometimes compared with concentrations previously assessed in other whole-insect novel foods. For dioxins and related compounds, some applications included direct analysis of the novel food, whereas others relied partly on feed-monitoring results or published data on whole insects. Most pesticide analytes were below the LODs or LOQs; where residues were quantified, reference values for other commodities, including Codex Alimentarius values in some assessments, were used for interpretation. Such reference values provided contextual benchmarks but did not constitute insect-specific MRLs. The distinction between product-specific novel food specifications and generally applicable regulatory limits is discussed further in Section 6.2.

Information on VMP and other pharmacologically active substances was generally provided as part of the description of the production process. Where this information was reported, applicants stated that antimicrobials, antiparasitic agents, VMP, growth hormones, or related substances were not intentionally used during insect rearing or processing. These declarations documented production practices but did not constitute analytical evidence confirming the absence of residues in the final novel foods.

Biogenic amines were assessed in several applications, although the compounds analysed and the approaches used for interpretation differed. Reported analytes included histamine, tyramine, putrescine, cadaverine, tryptamine, 2-phenylethylamine, spermidine, and spermine. Histamine concentrations were compared, for contextual purposes, with the food safety criterion established for specified fishery products [121], whereas, in the absence of generally applicable legal MLs for most biogenic amines, measured concentrations were contextualised using occurrence data from other foods. EFSA noted that biogenic amines may originate from endogenous biosynthesis, feed materials, the insect-associated microbiota, processing, or storage. In some applications, additional microbiological investigations, including analyses for *Pseudomonas aeruginosa*, were requested to explore possible explanations for their occurrence; the results did not indicate that this bacterium accounted for the measured concentrations.

Applicants also submitted stability data covering the proposed storage conditions and shelf life. Depending on the product, chemical stability was evaluated using indicators of lipid hydrolysis and oxidation, including acid value, free fatty acid content, peroxide value, p-anisidine value, and total oxidation (Totox) value. Broader stability assessments also included biogenic amines and microbiological parameters in selected applications. In several assessments, the measured parameters remained within the proposed specifications and supported the proposed shelf life. In others, interpretation was constrained by the use of different batches at different time points, limited numbers of batches, insufficient sampling intervals, or variability in lipid-degradation indicators. These limitations reduced the extent to which stability could be evaluated over the full proposed shelf life.

These differences are illustrated by the assessments of *A. domesticus* powder and frozen and dried formulations from whole yellow mealworm (*T. molitor larva*). For *A. domesticus* powder, EFSA considered the stability data from five independently produced batches analysed after 12 months sufficient to support the proposed shelf life [119]. By contrast, in the assessment of frozen and dried formulations from whole yellow mealworm, the oxidative-stability data for the powder at the beginning and end of storage were obtained from different batches, and p-anisidine values showed high variability. This limited the assessment of changes in oxidative stability over time [110].

Insect novel foods were proposed as frozen products, pastes, dried products, and powders and for use across a broad range of FoodEx2-defined food categories, with maximum use levels specified for each category. Where processing under the proposed conditions of use could affect safety, applicants submitted studies in selected intended-use matrices, including biscuits, cereal bars, pasta, soups, sausages, and meat analogues.

Processing-related contaminants assessed in intended-use matrices varied among applications and included acrylamide, furan and its derivatives, free 2-MCPD and 3-MCPD and/or their fatty acid esters, glycidyl fatty acid esters, PAHs, and heterocyclic aromatic amines. Results were interpreted against the analytical LODs or LOQs and, where applicable, acrylamide benchmark levels or MLs established for other food categories. Some studies also assessed oxidative stability and microbiological parameters.

The interpretative value of these studies varied considerably. In some opinions, foods containing the insect NF were compared with corresponding control products manufactured without the NF, allowing a more direct assessment of whether NF incorporation influenced contaminant formation. In the assessment of *A. domesticus* powder, selected intended-use foods containing the novel food were evaluated alongside corresponding control products prepared without it. Processing contaminants were examined in pasta, lipid oxidation in biscuits, and microbiological stability in cake [119]. By contrast, in the assessment of frozen and dried formulations of whole yellow mealworm, EFSA considered the evidence on processing-generated contaminants in the intended-use matrices insufficient because appropriate control samples were absent or inadequately characterised, or too few control samples were analysed [115].

More broadly, limited sample numbers, incomplete coverage of the proposed processing conditions, and variation in study design constrained the extent to which observed contaminant concentrations could be attributed specifically to the insect ingredient. Findings obtained from a limited number of test products could not necessarily be generalised across all proposed food categories, formulations, and processing conditions. These limitations are particularly relevant for insect NFs intended for incorporation into diverse products subjected to substantially different thermal and technological processes.

Regardless of the results or limitations of these studies, composite foods containing insect novel foods remain subject to the horizontal regulatory requirements applicable to the relevant final food category. Compliance of an authorised insect NF with its product-specific specifications does not replace the obligation to comply with applicable contaminant limits, pesticide-residue requirements, requirements for process contaminants, and other food safety provisions governing the final product.

Overall, the EFSA opinions provide detailed, product- and use-specific premarket assessments of insect-derived novel foods produced under defined conditions and intended for specified uses. The compositional, contaminant, stability, and intended-use data support the safety assessment and specifications of the individual NFs. However, differences in analytical coverage, study design, product formulation, processing conditions, and proposed uses limit direct comparisons among opinions. The dossier evidence therefore provides valuable insight into the hazards considered during premarket assessment but cannot, by itself, establish representative occurrence patterns in marketed products or generally applicable contaminant limits for edible insects as a food category. These data characterise the specific novel foods, production processes, and batches assessed in the respective applications and should not be interpreted as representative occurrence data for other insect products or for the wider edible-insect market.

### 6.2. Current Maximum Levels and Maximum Residue Levels Applicable to Edible Insects and Their Limitations

Regulation (EU) 2023/915 establishes MLs for a wide range of chemical contaminants, including heavy metals, mycotoxins, plant toxins, process contaminants, persistent organic pollutants, and other environmental contaminants in specified food categories [29]. However, it does not establish dedicated MLs for edible insects. Authorisations for individual insect NFs may include specifications for selected contaminants, primarily Cd and Pb, selected mycotoxins, and dioxins and dioxin-like PCBs. The product-contaminant-related chemical specifications relevant to the present review currently established for authorised insect-derived novel foods are summarised in Table 3 together with their legal basis. These specifications apply only to the respective authorised NF and do not constitute generally applicable MLs for edible insects as a food category [12]. The parameters included in these specifications and the evidence used to assess them vary among authorisations according to the insect species, formulation, production process, and data submitted for the individual NF.

In the absence of dedicated insect-specific MLs, contaminant concentrations reported in insect NFs have frequently been compared with MLs or other regulatory values established for different food categories. Such comparisons provide useful context for interpreting analytical results but do not necessarily indicate that the corresponding limits are legally applicable to insects. They also do not directly account for differences among insect species, production systems, feeding practices, contaminant uptake and metabolism, or anticipated consumption patterns.

This issue is particularly relevant for heavy metals, for which limits established for other food categories are commonly used as comparative reference points. Although these values provide provisional benchmarks, their applicability to edible insects cannot be assumed because insect biology, feeding behaviour, and exposure pathways may influence contaminant uptake and accumulation.

The regulatory situation is different for pesticide residues. MRLs under EU pesticide legislation are established for defined commodities listed in Annex I to Regulation (EC) No 396/2005, which explicitly includes “Insects” within the group “Terrestrial invertebrate animals” [13]. Edible insects may be exposed to pesticide residues through feed materials or other environmental sources, but under Article 18(1), pesticide residues in insects must comply with the applicable MRLs established under the Regulation. Where no specific MRL is established for the relevant pesticide–commodity combination, Article 18(1)(b) provides a default MRL of 0.01 mg/kg, subject to the other provisions of the Regulation [13]. Consequently, the absence of an MRL established specifically for an individual edible-insect species does not imply that no legally applicable MRL exists. Values established for unrelated commodities or by the Codex Alimentarius may be used as contextual comparison benchmarks in research studies but should not be presented as the applicable EU legal limit.

The applicability of limits for contaminants may also depend on the final food in which the insect NF is incorporated. Foods containing insect ingredients remain subject to the horizontal regulatory requirements applicable to the final food category, including relevant contaminant MLs, pesticide-residue provisions, and process-contaminant requirements. For pesticide residues, Article 20 of Regulation (EC) No 396/2005 specifies the application of MRLs to processed and/or composite foods where no specific MRL has been established for the processed or composite product [13]. A distinction must therefore be maintained between specifications applying to the authorised insect NF and regulatory limits applying to the resulting composite food.

Overall, the current framework combines specifications established through NF authorisations with horizontal contaminant and pesticide-residue legislation. However, it does not provide a harmonised set of insect-specific MLs or MRLs, and the interpretation of contaminant concentrations frequently relies on comparisons with values established for other food categories. This limits the consistency with which contaminant data can be interpreted across edible-insect products. Recent legal analysis similarly concluded that, although general EU food and feed legislation applies to farmed insects, existing legal limits do not consistently account for insect-specific production and exposure conditions [64]. The absence of a dedicated insect-specific limit should not, however, be interpreted in itself as a regulatory deficiency, nor does it imply that a separate insect-specific ML or MRL is necessary for every contaminant. Where horizontal requirements or product-specific specifications adequately address a chemical hazard, additional insect-specific limits may not be warranted. The relevant regulatory question is therefore whether the existing provisions provide a sufficiently clear and scientifically appropriate basis for monitoring, interpretation, and enforcement for the contaminant and product concerned.

### 6.3. Post-Market Monitoring and Occurrence-Data Gaps

Beyond premarket authorisation, Regulation (EU) 2015/2283 does not require routine post-market monitoring for NFs as a category [11]. Instead, the Commission may impose post-market monitoring requirements for an individual NF, where justified for food-safety reasons and taking into account EFSA’s opinion. Where applicable, these requirements are included in the entry for that NF in the Union list [11]. Authorised insect NFs also remain subject to the general official-control framework applicable to foods in the EU [129].

Thus, legal mechanisms exist for product-specific post-market monitoring and general regulatory control, but they do not in themselves ensure the systematic generation of comparable occurrence data across commercially available edible insects and insect-based products. More coordinated post-market data collection would help determine whether findings from novel food dossiers and controlled studies are representative of products available to consumers and would provide a stronger basis for exposure assessment and regulatory interpretation.

## 7. Research Gaps and Future Directions

Evidence on the chemical food safety of edible insects remains heterogeneous and difficult to generalise across species, production systems, and insect-based products. Much of the available literature is based on environmental or bioindicator studies, controlled exposure experiments, or insects investigated for feed rather than commercially produced insects intended for human consumption. These evidence types should therefore be clearly distinguished when interpreting their relevance to food safety and potential consumer exposure.

A major research gap is the limited availability of comparable occurrence data for commercially produced edible insects and insect-based foods. Differences in sampling designs, analytical methods, reporting practices, and the products investigated restrict comparisons across studies. Data from NF dossiers are valuable for assessing specific authorised NFs, but they are generated under defined production conditions and generally involve a limited number of batches. They therefore cannot establish occurrence patterns across the wider market. More systematic and comparable post-market monitoring data are needed to determine whether findings from dossiers and experimental studies are representative of commercially available insect-based foods.

Representative consumption data are also needed to translate occurrence data into meaningful estimates of dietary exposure. Such data should distinguish among whole insects, insect powders, insect ingredients, and composite foods and account for consumption frequency, portion size, high-consumer scenarios, and exposure from other dietary sources.

Important mechanistic uncertainties remain regarding contaminant transfer, accumulation, metabolism, and transformation. Experimental studies indicate that some heavy metals and pesticide residues may accumulate under particular exposure conditions, whereas selected mycotoxins and VMPs may undergo metabolism, transformation, or excretion [40,42,56]. A central unresolved question is whether reductions in detectable parent compounds correspond to reductions in total toxicologically relevant residues. Addressing this question requires mass-balance studies covering substrates, insect biomass, excreta, metabolites, and other transformation products, together with their identification and toxicological evaluation. The co-occurrence of contaminants and the potential relevance of combined exposure also remain poorly characterised, and future studies should therefore consider realistic contaminant mixtures rather than focusing exclusively on individual compounds.

Evidence on processing-generated contaminants is similarly limited. Although acrylamide, furan and its derivatives, free MCPDs, MCPD fatty acid esters, PAHs, glycidyl fatty acid esters, and heterocyclic aromatic amines have been investigated, in some NF dossiers or insect-specific studies, the available data cover only a restricted range of formulations, processing conditions, and final food matrices. Primary insect-specific evidence is also scarce for potentially relevant hazards such as PAHs associated with smoking or grilling, nitrosamines, packaging migrants, and mineral-oil hydrocarbons. Limited sample numbers, inadequate or absent control products, and incomplete coverage of relevant processing scenarios restrict conclusions on whether insect ingredients influence contaminant formation. Future studies should include appropriate controls and processing conditions representative of both commercial production and domestic preparation to distinguish effects associated with the insect ingredient from those arising from the wider formulation and thermal treatment.

Regulatory coverage differs among chemical-hazard classes. Generally applicable insect-specific MLs are not established under Regulation (EU) 2023/915, whereas pesticide residues in insects are covered by Regulation (EC) No 396/2005 through specific MRLs or, where applicable, the default MRL provision [13,29]. Product-specific NF specifications and horizontal regulatory provisions should therefore be distinguished when interpreting chemical occurrence data. Particular uncertainty remains for emerging contaminants such as PFAS and BFRs and for VMP residues, for which the available evidence and regulatory coverage remain limited [53,68,69,130].

Future research should therefore prioritise systematic occurrence studies of commercially available products, representative consumption data for relevant population groups, controlled substrate-to-insect transfer experiments, and the identification and toxicological evaluation of metabolites and transformation products. Broader investigation of processing-related hazards across representative formulations and processing conditions is also needed. Harmonised sampling and reporting approaches, matrix-specific analytical validation, chemical speciation where toxicologically relevant, and consistent reporting of concentration basis, analytical recovery, LODs, and LOQs would improve comparability. Transfer studies should analyse both the rearing substrate and insect biomass to support a robust assessment of contaminant fate. Together, these data would provide a stronger basis for dietary-exposure assessment and risk characterisation. From a regulatory perspective, risk-based insect-specific limits, clearer commodity classifications, or guidance on the application of existing regulatory values should be considered where sufficient occurrence and exposure data demonstrate that the current framework does not provide a consistent basis for interpretation and enforcement.

## 8. Conclusions

Chemical food safety in edible insects cannot be assessed from substrate contamination, insect species, or parent-compound concentrations in isolation. The relevant unit of assessment is the full production chain because contaminant uptake and transformation within insects, together with processing and storage, determine the chemical profile reaching consumers. Commercial-product studies confirm the occurrence of several chemical hazards, including toxic elements, selected pesticide residues, mycotoxins, PFAS, and processing- or storage-related contaminants, but these studies remain limited in number, geographical coverage, and product representation. By contrast, much of the evidence on substrate-to-insect transfer, accumulation, metabolism, and transformation derives from controlled-exposure, feed-related, or environmental studies and should therefore be interpreted primarily as mechanistic evidence rather than as representative occurrence data for commercially marketed foods. Accordingly, evidence of contaminant occurrence or accumulation potential should not be equated with dietary exposure or consumer risk without product-specific occurrence, consumption, and toxicological information.

The EU NF framework provides product-specific pre-market safety assessment, complemented by applicable horizontal food-safety legislation. The main priorities are to generate representative market-occurrence and consumption data, clarify contaminant transfer and transformation under food-relevant production conditions, and evaluate processing-related hazards in commercially relevant products. These data would strengthen dietary-exposure assessment and support clearer risk-based regulatory interpretation where existing provisions require clarification or adaptation.

## Figures and Tables

**Table 2:** Insect-specific studies investigating processing-related contaminants in insect-based foods.

Insect Ingredient	Final Food Product	Processing Conditions and/or Study Design	Investigated Contaminants	Insect-Free Control	Ref.
*Acheta domesticus* cricket flour: 100% in one flour sample and 8–20% in the remaining insect-based products	Commercial cricket flours, bars and crackers	Analytical method development and validation; 11 commercial insect-based products analysed; commercial manufacturing conditions NR	Acrylamide; furfural; 5-methylfurfural; 5-hydroxymethylfurfural (HMF)	No matched insect-free control; three commercially available conventional (non-insect) cereal bars were analysed for comparison	González-Gómez et al. [77]
Cricket flour (species NR)	Commercial insect bar containing cricket flour, apple, cinnamon and caramel	Analytical method development and validation; market-purchased commercial products analysed; commercial manufacturing conditions NR	5-Hydroxymethylfurfural (HMF)	No matched insect-free control; two commercially available conventional (non-insect) cereal bars were analysed for comparison	González et al. [78]
*Acheta domesticus* powder, added at 5, 10 or 15% (*w*/*w*)	Salty crackers prepared with white or whole-grain wheat flour	Baked at 180 °C for 25, 35 or 45 min	Acrylamide; 2-MCPD and 3-MCPD fatty acid esters; glycidyl fatty acid esters	Yes; corresponding 0% cricket-powder crackers	Hradecka et al. [79]
*Acheta domesticus* flour; untreated or fermented with *Lactiplantibacillus plantarum* No. 122; 10, 20, or 30% inclusion	Wheat bread	Untreated or fermented cricket flour incorporated into bread; baked at 220 °C for 25 min	Acrylamide	Yes; wheat-bread control without cricket flour; corresponding untreated-cricket-flour breads were used for comparison with fermented formulations	Bartkienė et al. [80]

Note: Commercial-product studies without matched formulation controls provide occurrence data but do not allow direct attribution of measured contaminant concentrations to the insect ingredient. HMF, 5-hydroxymethylfurfural; MCPD, monochloropropanediol; NR, not reported.

**Table 3:** Product-specific contaminant-related chemical specifications established for authorised insect-derived novel foods in the European Union.

Authorised Insect-Derived Novel Food	Product Form(s)	Product-Specific Contaminant-Related Chemical Specifications	Legal Basis
*Tenebrio molitor* larva (yellow mealworm)	Dried larva, whole or in powder form	Pb ≤ 0.075 mg/kg; Cd ≤ 0.1 mg/kg; Total aflatoxins (B1 + B2 + G1 + G2) ≤ 4 µg/kg; Aflatoxin B1 ≤ 2 µg/kg; DON ≤ 200 µg/kg; OTA ≤ 1 µg/kg	Commission Implementing Regulation (EU) 2021/882 [122]
*Tenebrio molitor* larva (yellow mealworm)	Frozen, dried and powder forms	Frozen: Pb ≤ 0.01 mg/kg; Cd ≤ 0.05 mg/kg; Dried/powder: Pb ≤ 0.075 mg/kg; Cd ≤ 0.1 mg/kg; All forms: total aflatoxins (B1 + B2 + G1 + G2) ≤ 4 µg/kg; aflatoxin B1 ≤ 2 µg/kg; DON ≤ 200 µg/kg; OTA ≤ 1 µg/kg; Sum of dioxins and dioxin-like PCBs (UB, WHO2005 PCDD/F-PCB-TEQ) ≤ 0.75 pg/g fat	Commission Implementing Regulation (EU) 2022/169 [123]
*Tenebrio molitor* larvae (yellow mealworm)	UV-treated powder of whole larvae	Pb ≤ 0.02 mg/kg; Cd ≤ 0.1 mg/kg; Hg ≤ 0.005 mg/kg; As ≤ 0.05 mg/kg; Aflatoxin B1 ≤ 2 µg/kg; Total aflatoxins (B1 + B2 + G1 + G2) ≤ 4 µg/kg; DON ≤ 200 µg/kg; OTA ≤ 1 µg/kg; PCDDs/F + PCB TEQ ≤ 0.75 pg/g fat	Commission Implementing Regulation (EU) 2025/89 [124]
*Locusta migratoria* (migratory locust)	Frozen, dried and powder forms	Pb ≤ 0.07 mg/kg; Cd ≤ 0.05 mg/kg; Total aflatoxins (B1 + B2 + G1 + G2) ≤ 4 µg/kg; Aflatoxin B1 ≤ 2 µg/kg; DON ≤ 200 µg/kg; OTA ≤ 1 µg/kg; Sum of dioxins and dioxin-like PCBs (UB, WHO2005 PCDD/F-PCB-TEQ) ≤ 1.2 pg/g fat	Commission Implementing Regulation (EU) 2021/1975 [125]
*Acheta* *domesticus* (house cricket)	Frozen, dried and powder forms	Pb ≤ 0.05 mg/kg; Cd ≤ 0.06 mg/kg; Total aflatoxins (B1 + B2 + G1 + G2) ≤ 4 µg/kg; Aflatoxin B1 ≤ 2 µg/kg; DON ≤ 200 µg/kg; OTA ≤ 1 µg/kg; Sum of dioxins and dioxin-like PCBs (UB, WHO2005 PCDD/F-PCB-TEQ) ≤ 1.25 pg/g fat	Commission Implementing Regulation (EU) 2022/188 [126]
*Acheta* *domesticus* (house cricket)	Partially defatted powder	Cyanide ≤ 5 mg/kg; Pb ≤ 0.1 mg/kg; Cd ≤ 0.025 mg/kg; Total aflatoxins (B1 + B2 + G1 + G2) ≤ 0.4 µg/kg; DON ≤ 200 µg/kg; OTA ≤ 1 µg/kg; Sum of dioxins and dioxin-like PCBs (UB, WHO2005 PCDD/F-PCB-TEQ) ≤ 1.25 pg/g fat	Commission Implementing Regulation (EU) 2023/5 [127]
*Alphitobius* *diaperinus* larvae (lesser mealworm)	Frozen, paste, dried and powder forms	Pb ≤ 0.1 mg/kg; Cd ≤ 0.05 mg/kg; Total aflatoxins (B1 + B2 + G1 + G2) ≤ 4 µg/kg; Aflatoxin B1 ≤ 2 µg/kg; DON ≤ 200 µg/kg; OTA ≤ 1 µg/kg	Commission Implementing Regulation (EU) 2023/58 [128]

Note: Only contaminant-related chemical specifications relevant to this review are shown; compositional, nutritional, physicochemical, stability-related, and microbiological specifications are not included. The values presented are product-specific specifications established for the respective authorised novel foods and should not be interpreted as generally applicable maximum levels (MLs) or pesticide maximum residue limits (MRLs) for edible insects as a food category. UB, upper bound; DON, deoxynivalenol; OTA, ochratoxin A; TEQ, toxic equivalent.

## Data Availability

No new data were created or analysed in this study. Data sharing is not applicable to this article.

## Supplementary Materials

The following supporting information can be downloaded at: https://www.mdpi.com/article/10.3390/toxics14090774/s1, Supplementary Table S1: EFSA evidence.
